# Effects of the First 1000 Days Program, a systems-change intervention, on obesity risk factors during pregnancy

**DOI:** 10.1186/s12884-021-04210-9

**Published:** 2021-10-27

**Authors:** Meg Simione, Laura Moreno-Galarraga, Meghan Perkins, Sarah N. Price, Man Luo, Milton Kotelchuck, Tiffany L. Blake-Lamb, Elsie M. Taveras

**Affiliations:** 1 Division of General Academic Pediatrics, Massachusetts General Hospital for Children, 125 Nashua Street, Suite 860, Boston, MA 02114 USA; 2 Department of Pediatrics, Harvard Medical School, Boston, MA USA; 3 Department of Pediatrics, Complejo Hospitalario de Navarra, IdiSNA, Pamplona, Navarre Spain; 4 Department of Obstetrics and Gynecology, Massachusetts General Hospital, Boston, MA USA; 5 Kraft Center for Community Health Leadership, Massachusetts General Hospital, Boston, MA USA; 6 Department of Nutrition, Harvard T.H. Chan School of Public Health, Boston, MA USA

**Keywords:** Pregnancy, Prenatal care, Maternal health, Infant, Health behaviors, Obesity risk factors, Childhood obesity, Systems-approach

## Abstract

**Background:**

First 1000 Days is a systems-oriented program starting in early pregnancy lasting through the first 24 months of infancy focused on preventing obesity and related risk factors among low income, mother-infant pairs. The program was developed in partnership with stakeholders to create an infrastructure for system-wide change. It includes screening for adverse health behaviors and socio-contextual factors, patient navigation and educational materials to support behavior change and social needs, and individualized health coaching for women at highest risk of obesity and has been shown to reduce excess gestational weight gain for women who were overweight at the start of their pregnancy. The purpose of this study was to examine changes from the first to third trimester for women participating in the First 1000 Days Program.

**Methods:**

We collected information through self-administered questionnaires during the first and third trimester of gestation and from electronic health records relating to obesity risk factors. Measures collected included behavior (i.e., diet, physical activity and screen time) and psychosocial (i.e., anxiety) outcomes, as well as enrollment in Women, Infant, and Children (WIC) program. We examined the extent to which participation in the program was associated with changes in behaviors and psychosocial outcomes among women during pregnancy.

**Results:**

Women completed surveys at their initial and third trimester prenatal visits (*n* = 264). Mean age (SD) was 30.2 (5.51) years and 75% had an annual household income of <$50,000. Mean pre-pregnancy body mass index (BMI) was 27.7 kg/m^2^ and 64% started pregnancy with a BMI ≥ 25 kg/m^2^. In multivariable adjusted models, we observed decreases in intake of sugary-drinks (− 0.95 servings/day; 95% CI: − 1.86, − 0.03) and in screen time (− 0.21 h/day; 95% CI: − 0.40, − 0.01), and an increase in physical activity (0.88 days/week; 95% CI: 0.52, 1.23) from the first to third trimester. We also observed a decrease in pregnancy-related anxiety score (− 1.06 units; 95% CI: − 1.32, − 0.79) and higher odds of enrollment in Women, Infant, and Children (WIC) program (OR: 2.58; 95% CI: 1.96, 3.41).

**Conclusions:**

Our findings suggest that a systems-oriented prenatal intervention may be associated with improvements in behaviors and psychosocial outcomes during pregnancy among low-income mothers.

**Trial registration:**

ClinicalTrials.gov (NCT03191591; Retrospectively registered on June 19, 2017).

## Background

Obesity remains highly prevalent and is a major contributor to chronic disease and other adverse consequences [1, 2]. Socioeconomic and racial/ ethnic disparities continue to persist despite national prevention efforts [3, 4]. Some of the origins of maternal and childhood obesity are linked to the first 1000 days, a period from conception through the first 2 years of life and have life course impacts [5–7]. During pregnancy, behaviors, such as maternal diet and physical activity, maternal anxiety, and connection to resources affect excessive weight gain and postpartum weight retention [8, 9]. As highlighted by the World Health Organization [10], this period represents a critical time for health-promotion interventions to prevent maternal and childhood obesity.

Intervention efforts focused on the first 1000 days have shown improvement in behaviors, psychosocial outcomes, and utilization of the Special Supplemental Nutrition Program for Women, Infants and Children (WIC) resulting in improved outcomes for women and their children [11]. These studies have predominately targeted individual-level of change [12–14], while few interventions have focused on a broader context of change using a systems-level approach. A systems-level approach brings together a diverse group of stakeholders to create an infrastructure that promotes change across clinical and public health systems. In addition, few studies have been designed to support women and infant from low-income households as they may be more vulnerable than their peers to socio-contextual factors that influence behaviors, psychosocial status, and connection to resources. The First 1000 Days Program was developed in conjunction with stakeholders to build an infrastructure for system-wide change for obesity prevention during the antenatal and postpartum periods [15, 16]. The program addresses clinical, behavior, and socio-contextual factors contributing to excess weight gain during pregnancy and during the first 2 years of life and has been shown to reduce excess gestational weight gain for women who were overweight at the start of their pregnancy [16].

The purpose of this study is to examine behavior (i.e, diet, screen time, physical activity), psychosocial (i.e., anxiety), and WIC program enrollment changes from the first to third trimester for low-income women at high risk for obesity participating in the systems-oriented First 1000 Days Program in the greater Boston area. We hypothesized that program participation would lead to improvements in behavioral and psychosocial risk factors and promote the use of social support services during pregnancy.

## Methods

### Study overview

Women enrolled in this study were participants in The First 1000 Days Program, a systems-level initiative that engages stakeholders across clinical and public health sectors to reduce the prevalence of obesity and obesity risk factors among mother-infant pairs who are low-income by addressing the levels of individual, family, and socio-contextual factors that hinder progress in obesity prevention. The system-wide intervention begins when women initiate prenatal care in their first trimester of pregnancy and offers support for mothers, their partners, and eventually the mother-partner-infant triads, throughout pregnancy and the child’s first 24 months. The conceptual framework, intervention design, evaluation methods, and primary results have been described in detail elsewhere [15, 16]. A subset of women completed surveys in their first and third trimesters of prenatal visits to examine individual-level changes in behavior and psychosocial outcomes. For this secondary analysis, a quasi-experimental pre-post design was used to evaluate the changes in the behavior and psychosocial outcomes from the first to third trimester visit among participants.

### Eligibility and recruitment

**Fig. 1:**
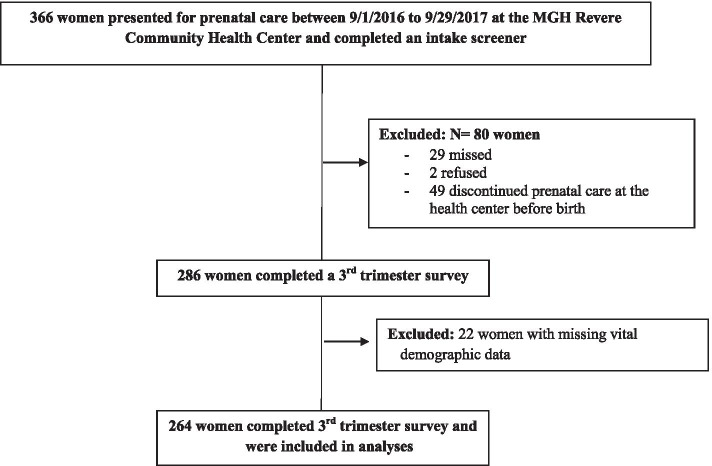
Participant flow diagram for women participating in The First 1000 Days Program during pregnancy

The First 1000 Days Program was offered to women who initiated prenatal care between August 2016 and September 2017 in three community health centers in Boston, Revere, and Chelsea, Massachusetts that serve predominantly low-income, racially/ethnically diverse populations. Upon completion of the intake survey and informed consent, women were considered to be enrolled in the program. The intake survey was administered to women at their first prenatal care visit which for most women was during the first trimester. Of the 366 women who completed a first trimester intake survey, 286 (78%) also completed a third trimester survey, and 264 (72%) women were included in the analyses as 22 women had missing vital demographic data (Fig. 1). The First 1000 Days study protocol was approved by Mass General Brigham Institutional Review Board and registered retrospectively at ClinicalTrials.gov (NCT03191591).

### Program components in pregnancy

The First 1000 Days Program has multiple components that aim to improve primary and secondary prevention of obesity. The program components that have been previously described in detail and include: staff and provider training emphasizing obesity prevention efforts; clinical decision support tools to track gestational weight gain; first prenatal visit universal screening for health behaviors and socio-contextual factors; patient navigation focused on healthy behavior change, social needs, and clinical and public health services; and health coaching for women at high risk of obesity [15, 16].

**Fig. 2:**
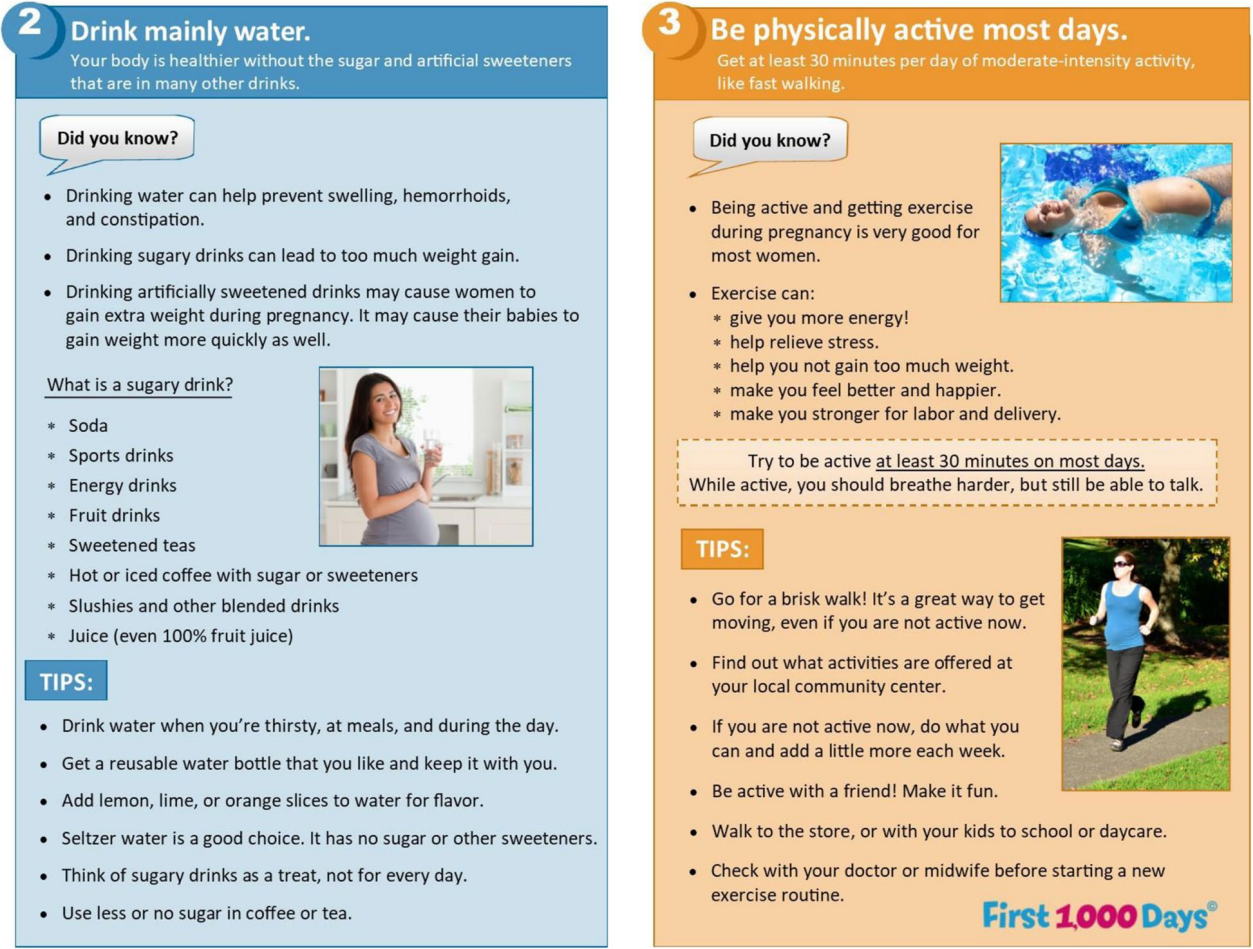
Examples of The First 1000 Days Programpregnancy booklet supporting behavioral changes

During pregnancy, the program focused on five behavior targets including: eating a balanced nutrition plan; drinking predominantly water and avoiding sugary-drinks; being physically active; getting recommended amounts of sleep; and reducing stress through social supports. Information regarding the behavior targets was delivered through printed materials including posters hanging in health centers and public health offices and individual booklets provided to patients. Booklets were available in English, Spanish, Vietnamese, and Arabic and contained customizable sections for gestational weight gain recommendations and behavior changed goal setting (Fig. 2). Women could also enroll in a text-messaging program to provide behavior change support and education and received 2–3 text messages during their pregnancy. Short informational videos (Vidscrips®) were also created in English and Spanish and available to women and their partners. The videos reinforced the behavior targets of the program, answered commonly asked questions, and provided recommendations.

### Data collection and outcome measurements

Information was collected through self-administered questionnaires during the first and third trimester of gestation and from electronic health records (EHR). Measures collected included behavior (i.e., diet, physical activity and screen time) and psychosocial outcomes, as well as WIC program enrollment. Surveys were available in English, Spanish, and Arabic.

Dietary behaviors, including fruit and vegetable, sugary-drinks, and fast food consumption were evaluated by asking women, “During the past 7 days, on average, how often did you eat …?”. Women responded by selecting: never; once per week, 2–4 times per week, nearly daily, 2–4 times per day, or 5 or more times per day. The fruit and vegetable consumption question included fresh, cooked, canned, or frozen fruits or vegetables and excluded juices or dried fruits; mean consumption was measured in times per day. The sugary-drink question included fruit-flavored drinks, juice from concentrate, punch, Kool-Aid, soda, sports drinks, sweet tea or coffee drinks, and sweetened milks; mean consumption was measured in beverages consumed weekly. The fast-food question asked about eating from a fast food restaurant; mean consumption was measured in weekly consumption. The items are from a validated food frequency questionnaire and have been previously used during pregnancy [17, 18].

To evaluate physical activity, women answered, “During the past 7 days, on how many days were you physically active for a total of at least 30 minutes per day? Add up all the time you spent in any kind of physical activity that increased your heart rate and made you breathe hard some of the time.” Responses to this question ranged from 0 to 7 days per week. This question was adapted from the Youth Risk Behavior Survey [19].

To measure screen time, women were asked, “During the past 7 days, on average, how many hours per day did you usually spend watching TV or videos. Include time spent watching on a television, computer, phone or tablet.” Women selected: never, < 1 h per day, 1, 2, 3, 4 or 5 h per day, and 6 or more hours per day. This question was adapted from the Nurses’ Health study [20].

We assessed pregnancy-related anxiety using the Pregnancy Anxiety Score, which focused on 5 topics: the extent of worry or concern about their health during pregnancy, about their baby’s health normal growth and developing, about losing the baby, about having a hard labor or difficult delivery, and about taking care of a new baby [21, 22] Possible responses to these questions were: never concerned, sometimes, most of the time, and almost all of the time concerned. A score was calculated by summing each of the five questions with a possible range from 0 to 20. Higher scores indicate a greater extent of worry or concern. If more than two questions were missing, a score was not calculated.

WIC program enrollment was offered to participants during pregnancy who reported they were not enrolled and met the income level criteria. To evaluate the number of women receiving WIC program support, we asked women, “Do you currently receive benefits from WIC?”. Women responded by answering: yes, no, or unsure.

### Confounding factors

Based on the previous literature, we used covariates for adjusted analyses [7, 23, 24]. Socio-demographic variables collected in the first and third trimester survey were used and they included maternal age and race/ ethnicity. Pre-pregnancy body mass index (BMI) was collected from the EHR.

### Statistical analysis

Data from the EHR and survey results were merged to allow for analyses. We compared participants’ responses at baseline (first trimester of gestation) with results from third trimester surveys; each participant was measured twice, resulting in pairs of observations. We used repeated measures design, including the paired t-tests for quantitative variables and the McNemar’s test for qualitative variables in unadjusted models. We also used multivariable regression models to adjust for possible confounders, including maternal age, race/ethnicity and pre-pregnancy BMI. Linear quantile mixed models were applied for the continuous behavior outcomes and mixed-effect linear models were applied for the continuous psychosocial outcome, with a time predictor to indicate the time points of first and third trimesters. The models accounted for clustering of observations within individuals. The adjusted median and mean differences between first and third trimester and 95% confidence intervals (CI) were calculated. For the dichotomous outcome, WIC enrollment, we applied logistic regression using generalized model, fitted with generalized estimating equation to address the repeated measurements. The adjusted odds ratio (OR) and 95% CI were generated for the WIC enrollment outcome. We performed analyses on complete cases and excluded participants with missing values in predictor, outcome, or covariates. A 2-sided alpha level of 0.05 was used to test for statistical significance in all analyses. Analyses were performed in RStudio 3.5.1 and SAS 9.4 (SAS institute, Cary, NC).

## Results

Of the 286 women who completed a survey at their initial and third trimester prenatal visit, 264 were included in final analyses. Baseline demographic characteristics of participants are summarized in Table 1. Women were a mean (SD) age of 30.8 (5.51) years and initiated their first prenatal care visit at a mean (SD) gestational age of 10.4 (4.65) weeks. Women had a mean (SD) pre-pregnancy BMI of 27.7 (6.43) kg/m^2^ and 64% started pregnancy with a BMI ≥ 25 kg/m^2^. At the first trimester visit, 33% of women were enrolled in WIC. 220 (83%) women received patient navigation and/or health coach phone-calls and booklets, 41(16%) received booklets only, and 3 (1%) did not receive a phone-call or booklet.

**Table 1:** Baseline characteristics of 264 women participating in the First 1000 Days Program

Maternal Characteristics at First Trimester Visit	N (%) or Mean (SD)
Gestational age at intake, weeks, mean (SD)	10.4 (4.65)
Maternal age at intake, years, mean (SD)	30.8 (5.51)
Parity, mean (SD)	1.98 (0.92)
Pre-pregnancy BMI, kg/m^2^, mean (SD)	27.7 (6.43)
Maternal pre-pregnancy weight status, n (%)	
Underweight	6 (2.3)
Healthy Weight	90 (34.1)
Overweight	94 (35.6)
Obese	74 (28.0)
Race/Ethnicity, n (%)	
White, Non-Hispanic	118 (44.7)
Hispanic or Latino	82 (31.1)
Black, Non-Hispanic	12 (4.5)
Other	52 (19.7)
Preferred language, n (%)	
English	141 (53.6)
Arabic	54 (20.5)
Spanish	46 (17.5)
Other	22 (8.4)
Annual household income, n (%)	
Less Than $20,000	78 (31.6)
$20,000 – $50,000	108 (43.7)
Greater Than $50,000	61 (24.7)
Employment status, n (%)	
Employed Full-Time	104 (40.0)
Employed Part-Time	72 (27.7)
Not Employed, Not Looking For Work	48 (18.5)
Not Employed, Looking For Work	22 (8.5)
Student	14 (5.4)
Married, n (%)	107 (41.0)
Publicly insured or uninsured, n (%)	133 (50.4)
Born outside the United States, n (%)	114 (43.2)

The results of the multivariable models adjusted for confounders for the behavior, psychosocial, and WIC enrollment outcomes are shown in Table 2. In multivariable adjusted models, we observed statistically significant decreases in intake of sugary-drinks (− 0.95 servings/week; 95% CI: − 1.86, − 0.03) and in screen time (− 0.21 h/day; 95% CI: − 0.40, − 0.01), and an increase in physical activity (0.88 days/week; 95% CI: 0.52, 1.23) from the first to third trimester. We did not observe statistically significant changes between the first and third trimester for fruit and vegetable or fast-food intake. We also observed a decrease in pregnancy-related anxiety score (− 1.06 units; 95% CI: − 1.32, − 0.79) and higher odds of enrollment in WIC (OR: 2.58; 95% CI: 1.96, 3.41).

**Table 2:** Median and mean changes from first to third trimester prenatal visit among women participating in the First 1000 Days Program, *N* = 264

**Outcomes**	**Median (IQR) First Trimester Visit**	**Median (IQR) Third Trimester Visit**	**Adjusted Median Differences**^**a**^ **(95% CI)**	***p*-value**
***Behavioral Outcomes***				
Fruit and vegetables (daily servings)	1.00 (0.43, 3.00)	1.00 (0.43, 3.00)	0.08 (−0.09, 0.24)	0.36
Sugary-drinks (weekly servings)	3.00 (1.00, 7.00)	3.00 (1.00, 7.00)	**−0.95 (−1.86, − 0.03)**	**0.04**
Fast food (weekly servings)	1.00 (0.00, 1.00)	0.00 (0.00, 1.00)	−0.03 (− 0.32, 0.26)	0.82
Physical activity (days per week)^b^	2.00 (0.00, 4.00)	3.00 (2.00, 5.00)	**0.88 (0.52, 1.23)**	**< 0.001**
Screen time (hours per day)	2.00 (1.00, 3.00)	2.00 (1.00, 3.00)	**−0.21 (−0.40, − 0.01)**	**0.04**
***Psychosocial Outcomes***	**Mean (SD) First Trimester Visit**	**Mean (SD) Third Trimester Visit**	**Adjusted Mean Differences**^**a**^ **(95% CI)**	***p*-value**
Pregnancy anxiety total score^c^	9.30 (2.93)	8.22 (2.67)	**−1.06 (−1.32, −0.79)**	**< 0.001**
	**First Trimester Visit n(%)**	**Third Trimester Visit n(%)**	**Adjusted OR**^**a**^ **(95% CI)**	***p*-value**
The Special Supplemental Nutrition Program for Women, Infants, and Children (WIC) enrollment^d^	86 (33.3%)	141 (55.1%)	**2.58 (1.96, 3.41)**	**< 0.001**

*CI* Confidence interval, *IQR* Interquartile range (first quartile, third quartile)

^a^Adjusted estimates from longitudinal model. Adjusted for maternal age at intake, race/ethnicity, and pre-pregnancy BMI

^b^*N* = 260

^c^*N* = 261; Range from 0 to 20 with higher scores indicating greater extent of worry or concern

^d^*N* = 250

## Discussion

The First 1000 Days Program was created to build an infrastructure for system-wide change, and our findings reveal that the program is associated with improvements in behavior and psychosocial outcomes that aid in obesity prevention during antenatal and postpartum periods. In this study, we examined behavior, psychosocial, and community resource connection changes in women from low-income households who participated in the First 1000 Days Program. We found participation in the program was associated with a decrease in intake of sugary-drinks, screen time, and pregnancy-related anxiety score; and an increase in physical activity from the first to third trimester. Women also had higher odds of enrollment in WIC after program participation.

Similar to other interventions during pregnancy, the First 1000 Days Program showed improvement in dietary behaviors illustrating that a systems-level approach can be an effective method for promoting behavior change. Previous interventions have found improvements in overall dietary intake as measured via Healthy Eating Index and the Semi Quantitative Food Frequency Questionnaire [25–27], decreased consumption of processed foods [28], and increased consumption of fruits and vegetables [29]. We found a decrease in consumption of sugary-drinks that has been shown to be critical in reducing post-partum weight retention [30], but we did not see changes in fruit, vegetables, and junk food intake. Other reasons, such as neighborhood characteristics and food environment, despite our systems-level approach, may have continued to make changes in these dietary areas more challenging for women [31]. Although clinically meaningful differences in dietary behaviors during pregnancy to improve obesity prevention are unknown, studies have shown that diet quality decreases throughout pregnancy (for example, increase in sugary drink consumption) [32–34]. Patient characteristics including educational level have also been shown to be associated with diet quality during pregnancy [35]; given the characteristics of the population in this study, we would hypothesize women were at high risk of poor diet quality. The temporal change in diet quality during pregnancy is suggestive that the First 1000 Days Program showed promising results in overcoming usual pregnancy dietary trends.

We found a modest, yet positive program effect on physical activity and screen time, though findings from previous interventions that have targeted physical activity are equivocal and few interventions have targeted screen time [13, 26, 27, 29, 36]. A meta-analysis of 13 studies that examined the effectiveness of physical activity interventions during pregnancy found increases in metabolic equivalents and physical fitness [37]. Ainscough and colleagues [26] found a mean difference of 141.1 min per week of physical activity between the intervention and control groups in their trial that targeted individual-level change. Although measured differently and in different units, we also found an increase of physical activity of 0.87 days per week. By improving physical activity levels and sedentary behaviors, such as screen time, programs can help improve pregnancy and infant outcomes including gestational diabetes mellitus, excess gestational weight gain, maternal mental health, and infants born large-for-gestational-age [13, 26].

In addition to behavior changes, we also found that women decreased their anxiety levels, consistent with what has been demonstrated in the literature [14, 38]. A meta-analysis revealed that interventions during pregnancy from four randomized control trials were shown to decrease anxiety scores resulting in a pooled estimate of − 1.74 units, similar to our findings of − 1.06 units and thereby improving women’s mental health during the antenatal period. Another key component of the First 1000 Days Program was connecting women to community resources including WIC program, food banks, and social services. By connecting women to community resources and engaging those organizations as stakeholders, the program was able to influence organizational and community factors that impact maternal health.

As previously reported, the First 1000 Days Program has been shown to reduce excess gestational weight gain for low-income women who were overweight at the start of their pregnancy [16]. In addition, women who participated in the program were also satisfied with the program, believed it improved their health and well-being, and that it provided an appropriate amount of services [16]. The changes in behavior and mental health likely contributed to the reduction in excess gestational weight gain. Although several programs have demonstrated improvements in behaviors, mental health, and gestational weight gain, many of these studies have focused on the individual level [12, 39–41], whereas the First 1000 Days Program focused on system-wide change. The results of this current study demonstrate that a system-wide approach is associated with improved maternal behavior and psychosocial outcomes that support women during pregnancy, and advantages of this approach are the connections between clinical and public health programs that target individual-level behaviors and socio-contextual risk factors simultatneously [16, 42].

This study presents with several limitations. Because the First 1000 Days Program utilized a system-wide change approach, i.e., the program had multiple components, we were unable to discern which component impacted the most change. The pre-post quasi-experimental design without a comparison group is susceptible to temporal confounders, but this needs to be balanced with a pragmatic approach to real-world implementation. Because of our pragmatic approach to implementation, approximately 30% of eligible women were not included in the final analysis due to reasons such as changing site of care and missing vital demographics. In addition, the outcomes of interest were self-reported which may have introduced bias.

## Conclusions

We found that women who participated in the First 1000 Days systems-oriented maternal and infant obesity prevention program decreased their sugary-drink consumption, screen time, and pregnancy-related anxiety score; increased their physical activity; and increased enrollment in WIC from the first to third trimester. The changes in behaviors, psychosocial status, and connections to resources found in this study are critical to improving maternal and child health outcomes. The findings indicate that the system-wide First 1000 Days Program may be associated with improvements in maternal behavior and psychosocial outcomes for obesity prevention during the antenatal and postpartum periods for low-income women.

## Data Availability

The datasets used during the current study are available from the corresponding author on reasonable request.

## Abbreviations

WIC: The Special Supplemental Nutrition Program for Women, Infants and Children
BMI: Body mass index
EHR: Electronic health records
OR: Odds ratio
CI: Confidence Interval

## References

[1] Massetti GM, Dietz WH, Richardson LC (2017). Excessive weight gain, obesity, and cancer: opportunities for clinical intervention. JAMA..

[2] Mokdad AH, Ballestros K, Echko M (2018). The state of US health, 1990-2016: burden of diseases, injuries, and risk factors among US states. JAMA..

[3] Rossen LM, Schoendorf KC (2012). Measuring health disparities: trends in racial-ethnic and socioeconomic disparities in obesity among 2- to 18-year old youth in the United States, 2001-2010. Ann Epidemiol.

[4] Olds T, Maher C, Zumin S (2011). Evidence that the prevalence of childhood overweight is plateauing: data from nine countries. Int J Pediatr Obes.

[5] Voerman E, Santos S, Inskip H (2019). Association of gestational weight gain with adverse maternal and infant outcomes. JAMA..

[6] Mamun AA, Mannan M, Doi SAR (2014). Gestational weight gain in relation to offspring obesity over the life course: a systematic review and bias-adjusted meta-analysis. Obes Rev.

[7] Woo Baidal JA, Locks LM, Cheng ER, Blake-Lamb TL, Perkins ME, Taveras EM (2016). Risk factors for childhood obesity in the first 1,000 days: a systematic review. Am J Prev Med.

[8] Nehring I, Schmoll S, Beyerlein A, Hauner H, von Kries R (2011). Gestational weight gain and long-term postpartum weight retention: a meta-analysis. Am J Clin Nutr.

[9] Siega-Riz AM, Viswanathan M, Moos M-K (2009). A systematic review of outcomes of maternal weight gain according to the Institute of Medicine recommendations: Birthweight, fetal growth, and postpartum weight retention. Am J Obstet Gynecol.

[10] World Health Organization. Taking Action on Childhood Obesity Report. Geneva; 2018. https://apps.who.int/nutrition/publications/obesity/takingaction-childhood-obesity-report/en/index.html.

[11] Blake-Lamb TL, Locks LM, Perkins ME, Woo Baidal JA, Cheng ER, Taveras EM (2016). Interventions for childhood obesity in the first 1,000 days: a systematic review. Am J Prev Med.

[12] Thangaratinam S, Rogozinska E, Jolly K (2012). Effects of interventions in pregnancy on maternal weight and obstetric outcomes: Meta-analysis of randomised evidence. BMJ..

[13] Chan CWH, Au Yeung E, Law BMH. Effectiveness of physical activity interventions on pregnancy-related outcomes among pregnant women: A systematic review. Int J Environ Res Public Health. 2019;16(10). 10.3390/ijerph16101840. 10.3390/ijerph16101840 PMC6571580 31126153

[14] van Dammen L, Wekker V, de Rooij SR, Groen H, Hoek A, Roseboom TJ (2018). A systematic review and meta-analysis of lifestyle interventions in women of reproductive age with overweight or obesity: the effects on symptoms of depression and anxiety. Obes Rev.

[15] Blake-Lamb T, Arauz Boudreau A, Matathia S (2018). Strengthening integration of clinical and public health systems to prevent maternal-child obesity in the first 1,000Days: a collective impact approach. Contemp Clin Trials.

[16] Blake-Lamb T, Boudreau AA, Matathia S (2020). Association of the First 1,000 days systems-change intervention on maternal gestational weight gain. Obstet Gynecol.

[17] Blum RE, Wei EK, Rockett HR (1999). Validation of a food frequency questionnaire in native American and Caucasian children 1 to 5 years of age. Matern Child Health J.

[18] Miller SA, Taveras EM, Rifas-Shiman SL, Gillman MW (2008). Association between television viewing and poor diet quality in young children. Int J Pediatr Obes IJPO an Off J Int Assoc Study Obes.

[19] Brener ND, Kann L, McManus T, Kinchen SA, Sundberg EC, Ross JG (2002). Reliability of the 1999 youth risk behavior survey questionnaire. J Adolesc Health.

[20] Jung SJ, Winning A, Roberts AL (2019). Posttraumatic stress disorder symptoms and television viewing patterns in the nurses’ health study II: a longitudinal analysis. PLoS One.

[21] Rini CK, Dunkel-Schetter C, Wadhwa PD, Sandman CA (1999). Psychological adaptation and birth outcomes: the role of personal resources, stress, and sociocultural context in pregnancy. Health Psychol.

[22] Cheng ER, Rifas-Shiman SL, Perkins ME (2016). The influence of antenatal partner support on pregnancy outcomes. J Womens Heal.

[23] Deputy NP, Sharma AJ, Kim SY, Hinkle SN (2015). Prevalence and characteristics associated with gestational weight gain adequacy. Obstet Gynecol.

[24] Flegal KM, Carroll MD, Kit BK, Ogden CL (2012). Prevalence of obesity and trends in the distribution of body mass index among US adults, 1999-2010. JAMA..

[25] Dodd JM, Deussen AR, Louise J. A randomised trial to optimise gestational weight gain and improve maternal and infant health outcomes through antenatal dietary, lifestyle and exercise advice: The OPTIMISE randomised trial. Nutrients. 2019;11(12). 10.3390/nu11122911. 10.3390/nu11122911 PMC6949931 31810217

[26] Ainscough KM, O’Brien EC, Lindsay KL (2019). Nutrition, behavior change and physical activity outcomes from the PEARS RCT: An mHealth-supported, lifestyle intervention among pregnant women with overweight and obesity. Front Endocrinol (Lausanne).

[27] Dodd JM, Louise J, Cramp C, Grivell RM, Moran LJ, Deussen AR. Evaluation of a smartphone nutrition and physical activity application to provide lifestyle advice to pregnant women: The SNAPP randomised trial. Matern Child Nutr. 2018;14(1). 10.1111/mcn.12502. 10.1111/mcn.12502 PMC6866107 28836373

[28] Flynn AC, Seed PT, Patel N (2016). Dietary patterns in obese pregnant women; influence of a behavioral intervention of diet and physical activity in the UPBEAT randomized controlled trial. Int J Behav Nutr Phys Act.

[29] Dodd JM, Cramp C, Sui Z (2014). The effects of antenatal dietary and lifestyle advice for women who are overweight or obese on maternal diet and physical activity: the LIMIT randomised trial. BMC Med.

[30] Mahabamunuge J, Simione M, Hong B, et al. Association of sugar-sweetened beverage intake with maternal postpartum weight retention. Public Health Nutr. 2020:1–8. 10.1017/S1368980020005169. 10.1017/S1368980020005169 PMC8609823 33336643

[31] Cummins S, Macintyre S (2006). Food environments and obesity-neighbourhood or nation?. Int J Epidemiol.

[32] Forbes LE, Graham JE, Berglund C, Bell RC. Dietary change during pregnancy and women’s reasons for change. Nutrients. 2018;10(8). 10.3390/nu10081032. 10.3390/nu10081032 PMC6115730 30096771

[33] Skreden M, Bere E, Sagedal LR, Vistad I, Øverby NC (2015). Changes in beverage consumption from pre-pregnancy to early pregnancy in the Norwegian fit for delivery study. Public Health Nutr.

[34] Crozier SR, Robinson SM, Borland SE, Godfrey KM, Cooper C, Inskip HM (2009). Do women change their health behaviours in pregnancy? Findings from the Southampton Women’s survey. Paediatr Perinat Epidemiol.

[35] Hillier SE, Olander EK (2017). Women’s dietary changes before and during pregnancy: a systematic review. Midwifery..

[36] Hayes L, Mcparlin C, Kinnunen TI, Poston L, Robson SC, Bell R (2015). Change in level of physical activity during pregnancy in obese women: findings from the UPBEAT pilot trial. BMC Pregnancy Childbirth.

[37] Flannery C, Fredrix M, Olander EK, McAuliffe FM, Byrne M, Kearney PM (2019). Effectiveness of physical activity interventions for overweight and obesity during pregnancy: a systematic review of the content of behaviour change interventions. Int J Behav Nutr Phys Act.

[38] Bogaerts AFL, Devlieger R, Nuyts E, Witters I, Gyselaers W, Van den Bergh BRH (2013). Effects of lifestyle intervention in obese pregnant women on gestational weight gain and mental health: a randomized controlled trial. Int J Obes.

[39] Muktabhant B, Lawrie TA, Lumbiganon P, Laopaiboon M (2015). Diet or exercise, or both, for preventing excessive weight gain in pregnancy. Cochrane Database Syst Rev.

[40] Peaceman AM, Clifton RG, Phelan S (2018). Lifestyle interventions limit gestational weight gain in women with overweight or obesity: LIFE-moms prospective meta-analysis. Obesity..

[41] The International Weight Management in Pregnancy Collaborative Group (2017). Effect of diet and physical activity based interventions in pregnancy on gestational weight gain and pregnancy outcomes: meta-analysis of individual participant data from randomised trials. BMJ.

[42] Paul KH, Graham ML, Olson CM (2013). The web of risk factors for excessive gestational weight gain in low income women. Matern Child Health J.

